# Identification of CXCL12 as a key gene of trophoblast and endometrial stromal cell decidualization in pregnancy via scRNA-seq and experimental validation

**DOI:** 10.3389/fmolb.2026.1768015

**Published:** 2026-01-30

**Authors:** Xianli Sun, Haimei Yang, Zhe Leng, Wei Zhang, Zhou Jiang, Henglian Liu

**Affiliations:** 1 Department of Gynecology, Chongqing Traditional Chinese Medicine Hospital, Chongqing, China; 2 Department of Gynecology and Obstetrics, Women and Children’s Hospital, Qingdao University, Qingdao, China; 3 Department of Reproductive Medicine, Women and Children’s Hospital, Qingdao University, Qingdao, China; 4 Department of Gynecology and Obstetrics, Binzhou People’s Hospital Affiliated to Shandong First Medical University, Binzhou, China; 5 Attending Physician Maternal and Child Health Care and Family Planning Service Center of Shibei District, Qingdao, China

**Keywords:** CXCL12, CXCR4, decidualization, maternal-fetal interface, recurrent miscarriage

## Abstract

**Background:**

Recurrent miscarriage (RM) remains a significant clinical challenge due to its elusive pathogenesis. This study aimed to investigate the role of chemokine ligand 12 (CXCL12) at the maternal-fetal interface and its specific mechanism in RM, to provide an experimental basis for potential diagnostic and therapeutic strategies.

**Methods:**

We utilized single-cell RNA-sequencing (scRNA-seq) to distinct single-cell atlas at the maternal fetal interface in normal and RM samples. And we comparably analyzed the expression of CXCL12 of Syncytiotrophoblast/Extravillous Trophoblast (SCT/EVET) in normal and unexplained RM pregnancies at the early stage of gestation. A CXCL12 silencing and overexpression system was established in the HTR-8/SVneo cell line. Cell viability was determined by MTT assay. The horizontal migration ability of HTR-8 cells was determined by cell scratch assay, while the invasion ability was tested by transwell matrix gel invasion assay. Recombinant human CXCL12 was used to stimulate endometrial stromal cells to explore the role of CXCL12 in the process of endometrial decidualization.

**Results:**

Single-cell atlas at the maternal fetal interface in normal and RM samples showed 11 cell types, including DSCs and SCT/EVT cells. In SCT/EVT cells, CXCL12 was remarkably decreased in RM cases. CXCL12 overexpression significantly promoted the proliferation, migration, and invasion ability of HTR-8 cells, while silencing CXCL12 significantly inhibited the proliferation, migration, and invasion ability of HTR-8 cells. Recombinant human CXCL12 significantly promoted the decidualization process of endometrial stromal cells, while decidualization was inhibited by CXCR4-neutralizing antibodies to a certain extent.

**Conclusion:**

CXCL12 promotes the proliferation, migration, and invasion ability of trophoblast cells *in vitro* and promotes the decidualization of endometrial stromal cells. The downregulation of the CXCL12/CXCR4 axis may be involved in the occurrence and development of RM. Thus, this study provides experimental clues for exploring potential therapeutic approaches related to CXCL12.

## Introduction

Successful pregnancy requires cooperation between fetal-derived trophoblast cells and differentiated maternal cells. The complex development process also involves interactions between various factors including chemokines, cytokines, and growth factors, which collectively contribute to the maintenance of maternal immune tolerance until delivery. Among these factors, the unique chemokine network during pregnancy has received considerable attention in the last decade as a feature of a successful pregnancy (Caba et al., 2002). When the expression levels of chemokines and their receptors are abnormal, immune tolerance at the maternal–fetal interface may be disrupted, leading to failed embryo implantation or impaired placenta formation and thus pregnancy failure (Salamonsen et al., 2007; Hannan and Salamonsen, 2007).

Recurrent miscarriage (RM) occurs for many reasons. In addition to imposing an economic burden on women and their families, RM can seriously affect the physical and mental health of women. In recent years, studies have revealed a regulatory network of chemokines and their receptors among trophoblast cells, decidual stromal cells (DSCs), and decidual immune cells. This network plays a crucial role in regulating trophoblast cell proliferation and invasion, promoting neovascularization, and facilitating the recruitment, migration, and homing of decidual immune cells (Hanna et al., 2003; Schanz et al., 2011; Piao et al., 2012; Ren et al., 2012; Zheng et al., 2018; Red-Horse et al., 2005; Wu et al., 2005; Koopman et al., 2003). Both CXCL12 and its receptor CXCR4 are expressed in early pregnancy trophoblast cells, and the corresponding CXCL12/CXCR4 axis regulates the proliferative effects of trophoblast cells (Wu et al., 2004). CXCL12 can induce the chemotaxis of CD56^+^CD16^−^ natural killer (NK) cells from peripheral blood to decidual tissue (Wu et al., 2005), thereby maintaining immune tolerance at the maternal–fetal interface and allowing the pregnancy to proceed. Thus, CXCL12 plays a crucial role in early pregnancy.

In modern society, the increase in infertility each year has caused serious social and health problems, with one of the main causes being early pregnancy disorder (Tamrakar and Bastakoti, 2019). The establishment of early pregnancy involves uterine receptivity, embryonic implantation, and decidualization of the endometrium. These processes, if problematic, can have adverse chain effects throughout the pregnancy, leading to adverse pregnancy outcomes such as infertility, miscarriage, preterm birth, and preeclampsia (Hendriks et al., 2019; Hanson et al., 2017). Endometrial decidualization includes morphogenic, biochemical, and vascular changes driven by both oestrogen and progesterone receptors. The decidua has multiple functions, including providing sources of growth factors and cytokines that support embryonic development, exerting immunomodulatory roles during pregnancy, and regulating trophoblast invasion. The status of endometrial receptivity and embryo implantation are closely related to the decidualization process of endometrial epithelial cells and stromal cells, including the remodeling of epithelial cells and the proliferation and differentiation of stromal cells (Bhurke et al., 2016; Zhang S. et al., 2013). The hallmark of early endometrial remodeling in humans is decidualization-after differentiation, decidual cells acquire unique biochemical and cellular properties that enable them to support embryo implantation. Thus, the decidualization of the human endometrium is essential for a successful pregnancy.

However, the specific mechanisms regulating the decidualization process remain largely unknown. No studies have described the regulation of chemokines and their receptors during the decidualization of endometrial stromal cells (ESCs).

In a previous study (Sun et al., 2022), we confirmed that the CXCL12 expression level in peripheral serum was low for non-pregnancies and miscarriages but increased significantly after pregnancy. In addition, the low level of CXCL12 in peripheral blood and low expression of CXCR4 protein in the decidua were correlated with the occurrence of early spontaneous abortion, suggesting that CXCL12 is involved in the maintenance of normal pregnancy. This study further explored the specific mechanism of CXCL12 in the regulation of the maternal–fetal interface and its role in the occurrence and development of RM.

## Materials and methods

### Data collection

In this paper, a single cell dataset GSE214607 containing decidual and villi samples from five women who underwent elective termination of normal pregnancies without a history of miscarriages and three women with recurrent miscarriage (RM) were obtained by Gene Expression Omnibus (GEO) database (https://www.ncbi.nlm.nih.gov/geo/). RM inclusion criteria were as follows: fetal cardiac activity assessed by Doppler ultrasound showed no pulsation or cardiac arrest at 7–9 weeks of pregnancy, and a history of two or more unexplained abortions (Wei et al., 2022). This study utilized single-cell data from this dataset. As the datasets were obtained from public databases, no ethical approval was required.

### Analytical process for scRNA-seq data

The scRNA-seq data of GSE214607 were processed using the Seurat package to create a Seurat object. Cells were filtered based on the following criteria: genes detected <250 or >5000, and mitochondrial contamination >25%. The merged object was normalized, and the top 3,000 highly variable genes were identified. Principal component analysis (PCA) was performed using these genes to extract principal components (PCs). We performed dimensionality reduction through Uniform Manifold Approximation and Projection (UMAP), generating two-dimensional visualizations of cell clusters. We used the “Single R” R package for automated annotation of cell types to annotate the cell subgroups. Decidual stromal cells (DSCs) and Syncytiotrophoblast/Extravillous Trophoblast (SCT/EVT) were defined by literature (Wei et al., 2022). Then, we used CXCL12-related genes in SCT/EVT cell using the Seurat package to get the expression of CXCL12 in SCT/EVT cell and divided it into high-CXCL12 and low-CXCL12 groups based on the median level. Differentially expressed genes (DEGs) between high-CXCL12 and low-CXCL12 expression were screened out according to |log2 fold change (FC)| >1 and *p*-value <0.05. Volcano map was used to visualize DEGs. These genes were imported into the David 6.8 database for Gene Ontology (GO) and Kyoto Encyclopedia of Genes and Genomes Enrichment (KEGG) enrichment analyses. According to the P value <0.05, the significantly enriched gong energy or pathway was visualized.

### Bacterial culture and plasmid extraction

Plasmid extraction was performed using an endotoxin-free plasmid extraction kit (Mei5bio, Beijing, China). The concentration and purity of the extracted plasmids were measured by ultraviolet spectrophotometry (Thermo Fisher Scientific, Waltham, MA, USA). Plasmids with a sufficiently high concentration and purity were selected for subsequent transfection operation.

### Cell cultures of HTR-8/SVneo cells and ESCs

The human chorionic trophoblast cell line HTR-8/SVneo (Zhongqiao Xinzhou Biotechnology, Shanghai, China) and ESCs (Procell, Wuhan, China) were planted in T25 culture bottles at 37 °C and 5% CO_2_ in an incubator. The cell status was observed regularly, and the culture medium was changed once each 1–2 days. The cells were sub-cultured after reaching 70%–80% confluence.

### Cell transfection

HTR-8/SVneo cells were digested using 0.25% trypsin cell digestion solution to obtain a suspension of single cells. The cells were plated in 6-, 24-, or 96-well plates at 37 °C and 5% CO_2_. Transfection operations began when the cells grew to 50%–60% confluence. Lipofectamine 3,000 (Invitrogen, Carlsbad, CA, USA) was transfected with the CXCL12 plasmid, siRNA, and the corresponding empty plasmid and empty siRNA.

### Cell viability assay

HTR-8/SVneo cells were seeded in 96-well plates and grown in an incubator for 48 h after transfection with plasmids and siRNA. Cell viability was determined by MTT assay (Sangon Biotech, Shanghai, China). The absorbance value of each well was detected using a microplate reader (Thermo Fisher Scientific) at a wavelength of 490 nm.

### Cell scratch assay

HTR-8/SVneo cells were seeded in 6-well plates and incubated with plasmid and siRNA until the cells reached 100% confluence. A scratch was made in the middle of each well with a 200 μL pipette tip perpendicular to the plate. Cells that fell from the scratch were removed with precooled PBS buffer. Medium was then added to each well for continued growth in a constant-temperature incubator. At 24 h after the scratch, the migration rate of the cells in each group was calculated.

### Transwell assay

HTR-8/SVneo cells were seeded in 6-well plates and grown in an incubator for 24 h after transfection with the plasmid and siRNA. The upper Transwell chamber was coated using 200 μg/mL Matrigel matrix (BD Biosciences, NJ, USA). After the matrix gel solidified, the cell suspension was added to the upper Transwell chamber so that the number of cells in the upper chamber was 5 × 10^4^. RPMI complete medium containing 10% FBS was added to the 24-well plate under the Transwell chamber. Finally, the cells were incubated in a at 37 °C and 5% CO_2_ for 24 h. After incubation, the cells were washed, fixed, and stained with hematoxylin for 30 min. The number of cells in the Transwell chamber was counted under a light microscope and recorded. The invasion rate was calculated for each group and statistically analyzed.

### Decidualization of ESCs

Endometrial stromal cells were seeded in 6-well plates to which complete medium had been added. The experimental groups were as follows. The negative control group received no additional stimuli. The positive control group received 8-bromo-cAMP (Sigma, St Louis, MO, USA) to a final concentration of 0.05 mM and medroxyprogesterone (MPA, Sigma) to a final concentration of 1 μM. The CXCL12 group was treated with recombinant human CXCL12 with a final concentration of 20 ng/mL. The CXCL12+Anti-CXCR4 group received recombinant human CXCL12 to a final concentration of 20 ng/mL and CXCR4-neutralizing antibody with a final concentration of 10 μM. The cells were cultured in a incubator, and the cell morphology was observed under microscope on the fourth day. Subsequently, total proteins were extracted from all groups. To verify the degree of decidualization in each group, the protein expression levels of IGFBP-1, a marker of DSCs, were measured by immunoblotting and normalized by β-actin as the housekeeping gene.

### Western blot

Rinse cells twice with pre-cooled phosphate buffer solution (PBS, pH7.4). Tissue lysis using precooled high-strength RIPA (Yeason, Shanghai, China) containing 1% protease inhibitor (Yeason, Shanghai, China) and phosphatase inhibitor (Yeason, Shanghai, China). The lysis products were centrifuged at 2500 g at 4 °C for 20 min. Collected supernatant and added on loading buffer, water boil for 10 min. Determination of protein concentration in supernatant using BCA kit (Yeason, Shanghai, China). Equal amounts of proteins were separated by gel electrophoresis on a 12.5%SDS-PAGE and transferred onto a PVDF membrane (Merck Millipore, Germany). Membranes were blocked in 5% (wt/vol) instant skim milk for 1 h at room temperature. Membranes were incubated at 4 °C overnight with primary antibodies (SDF1 Antibody (Monmouth Junction, NJ, USA; 1:1000), IGFBP1 Antibody (Monmouth Junction, NJ, USA; 1:1000), Beta Actin Antibody (Monmouth Junction, NJ, USA; 1:10,000). Following incubation with the primary antibody, membranes were then washed and incubated with secondary antibodies (HRP-labeled Goat Anti-Mouse/rabbit IgG (H + L) 1:10,000). Protein-antibody complexes were detected using the ECL hypersensitive luminescence kit (Yeason, Shanghai, China), and relative quantification of the protein bands was performed using ImageJ software.

### Data analysis

Statistical analysis and mapping were completed using GraphPad Prism 9. The results of all statistical analysis are reported as mean ± standard error of the mean. Differences between two groups were analyzed by Student’s t-test. For comparisons among multiple groups, one-way ANOVA was applied after confirming normal distribution and homogeneity of variances, followed by Tukey’s honest significant difference (HSD) *post hoc* test for pairwise comparisons. Differences were considered statistically significant as *p* < 0.05. All basic experiments were performed more than three times.

## Results

### Single-cell atlas at the maternal fetal interface in normal and RM samples

**FIGURE 1:**
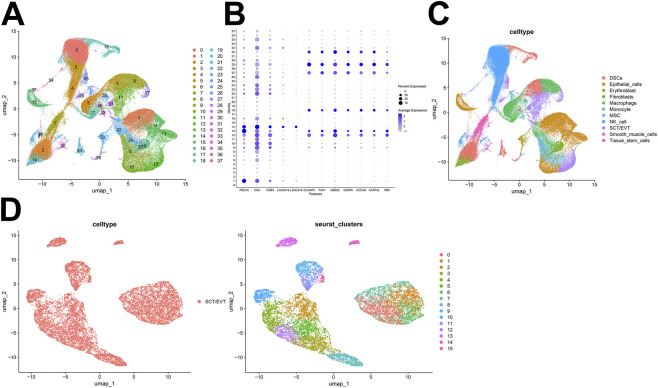
Single‐cell atlas at the maternal fetal interface in normal and recurrent miscarriage (RM) samples. **(A)** All cells are grouped into 38 clusters. **(B)** The bubble plots showed the expression levels of marker genes in each cluster. **(C)** Cell annotations for these clusters. **(D)** UMAP representation distinguishing 16 SCT/EVT cells subsets post-clustering: (Left) Cell annotations for SCT/EVT cells clusters; (Right) The UMAP showed the distribution of SCT/EVT cells clusters.

We obtained 16 human first-trimester decidual and villi samples from five normal patients and 3 RM patients using 10× Genomics, with decidual and villi samples collected from the same patient. Following computational quality control and graph-based clustering using the Seurat package, 112,528 high-quality cells were subjected to further analysis. These cells consisted of 36,219 cells from normal decidua and 25,582 cells from RM decidua, 25,303 cells from normal villi and 25,424 cells from RM villi. We performed cell cycle effect removal, normalization, dimensionality reduction, and clustering on the scRNA-seq data (Figure 1A). The bubble plots showed the expression levels of marker genes in each cluster (Figure 1B). After unsupervised graph-based analysis using Single R software, we automatically annotated the cell clusters and identified 11 cell types assigned on the basis of known marker genes and literature evidence, including DSCs and SCT/EVT cells (Figure 1C). Further analysis of the SCT/EVT cells subtype subset through PCA revealed 16 distinct subtypes (Figure 1D).

### Identification and functional enrichment analysis of DEGs related to CXCL12

**FIGURE 2:**
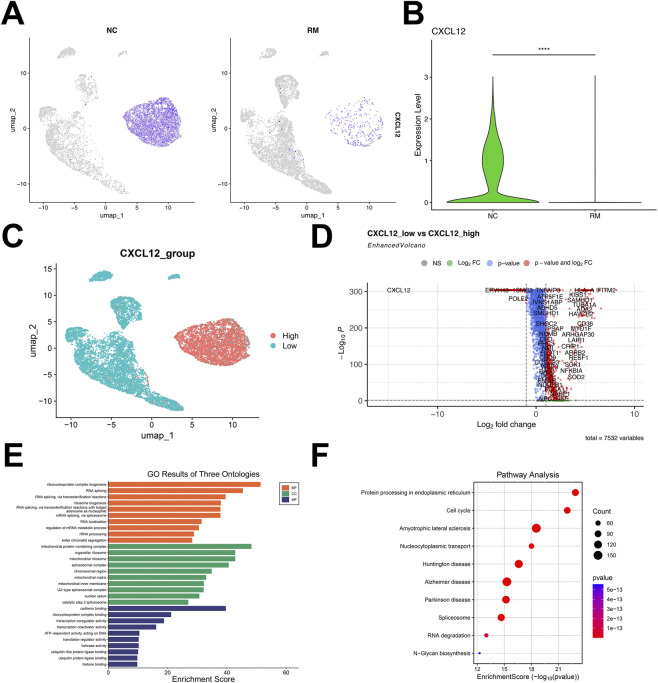
Identification and functional enrichment analysis of DEGs related to CXCL12. **(A)** UMAP visualization of the log-transformed, normalized CXCL12 expression in SCT/EVT cells subset in NC (left) and RM (right) patients. High expression is shown in purple, and low expression in gray. **(B)** Violin plots showing the differential expression of CXCL12 in NC and RM patients. **(C)** According to the CXCL12-related genes, the cells were divided into high-CXCL12 group and low-CXCL12 group, and the DEGs between the two groups were obtained. **(D)** Volcano plot showing the DEGs between high-CXCL12 group and low-CXCL12 group. **(E)** GO analysis of DEGs related to CXCL12. **(F)** KEGG analysis of DEGs related to CXCL12. ****p < 0.0001.

Based on the key role of CXCL12 in early pregnancy, we further analyzed the expression of CXCL12 in SCT/EVT cells in normal and RM cases. The results showed that CXCL12 in SCT/EVT cells was remarkably decreased in RM cases (Figures 2A,B). Next, based on the CXCL12 gene expression, subtypes were classified into high and low CXCL12 groups, depending on whether their average expression values were above or below the mean, respectively (Figure 2C). Then, the differential expression analysis of genes between high-CXCL12 and low-CXCL12 groups was carried out, and 7532 DEGs related to CXCL12 were obtained by setting *p* < 0.05 (Figure 2D). GO enrichment analysis of these CXCL12-related DEGs showed that they were enriched in sister chromatid segregation, cadherin binding, ubiquitin protein ligase binding (Figure 2E). KEGG enrichment analysis of these DEGs related to CXCL12 revealed that the main signaling pathways involved cell cycle, protein processing in endoplasmic reticulum and Nucleocytoplasmic transport (Figure 2F). These pathways or processes are associated with cell proliferation, migration or invasion.

### Overexpression of CXCL12 promotes cell growth

**FIGURE 3:**
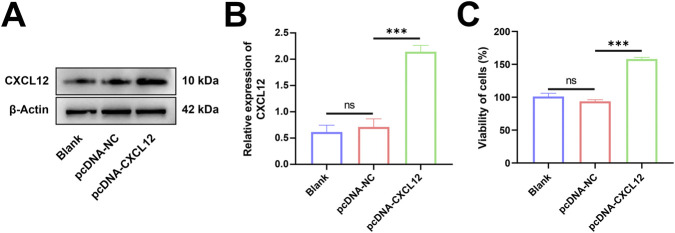
Overexpression of CXCL12 promotes cell growth. **(A)** Western blot analysis of CXCL12 expression in HTR-8 cells transfected with CXCL12 cDNA. **(B)** Statistical analysis was performed after repeating the experiment, and the CXCL12 protein expression of pcDNA-CXCL12 was significantly higher than that of pcDNA-NC. **(C)** The growth and proliferation of HTR-8 cells after CXCL12 cDNA transfection were compared with pcDNA-NC by MTT colorimetric assay. Ns, no significance; ***p < 0.001. Blank: blank control group; pcDNA-NC: empty control group; pcDNA-CXCL12: CXCL12 overexpression group.

To explore the role of CXCL12 in miscarriage, we transfected HTR-8 cells using a plasmid carrying CXCL12 cDNA. Western blotting confirmed that HTR-8 cells effectively overexpressed CXCL12 after being transfected with CXCL12 cDNA (Figure 3A). Statistical analysis was performed after repeated experiments, demonstrating that CXCL12 was significantly overexpressed in HTR-8 cells after CXCL12 cDNA transfection (Figure 3B; *p* < 0.05). The growth and proliferation of CXCL12-overexpressing HTR-8 cells were evaluated using MTT assay. The overexpression of CXCL12 significantly promoted the growth of HTR-8 cells compared with the pcDNA-NC (Figure 3C, *p* < 0.001).

### Overexpression of CXCL12 promotes cell migration and invasion

**FIGURE 4:**
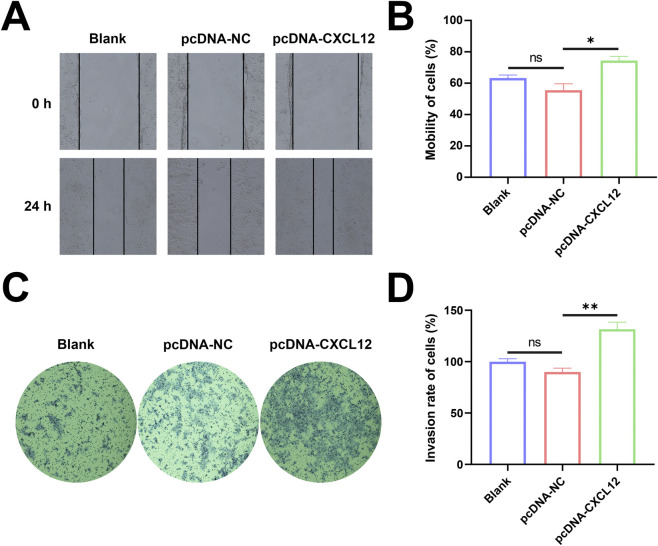
Overexpression of CXCL12 promotes cell migration and invasion. **(A)** The migration of HTR-8 cells after CXCL12 overexpression was detected by cell scratch assay (100×). **(B)** Cell mobility after 24 h was significantly upregulated in the CXCL12 overexpression group compared with the pcDNA-NC group. **(C)** Transwell invasion experiment detected the invasion of HTR-8 cells after CXCL12 overexpression (100×). **(D)** 24 h of cell mobility, significantly upregulated compared with the pcDNA-NC group. Ns, no significance; *p < 0.05; **p < 0.01. Blank: blank control group; pcDNA-NC: empty control group; pcDNA-CXCL12: CXCL12 overexpression group.

Next, we investigated whether CXCL12 regulates the migratory or invasive capacity of HTR-8 cells. Changes in the horizontal migration ability of HTR-8 cells after CXCL12 overexpression were determined by cell scratch assay. The overexpression of CXCL12 promoted the horizontal migration of HTR-8 cells, and the cell migration rate increased significantly in the CXCL12 overexpression group at 24 h after cell scratching (Figures 4A,B, *p* < 0.05). The invasive capacity of HTR-8 cells after CXCL12 overexpression were evaluated by invasion assay. The overexpression of CXCL12 significantly promoted HTR-8 cell invasion (Figure 4C), and the aggressiveness of HTR-8 cells overexpressing CXCL12 was significantly higher compared with the empty control group (Figure 4D, *p* < 0.01). These results indicate that the overexpression of CXCL12 promotes the migration and invasive ability of HTR-8 cells.

### Silencing of CXCL12 inhibits cell growth

**FIGURE 5:**
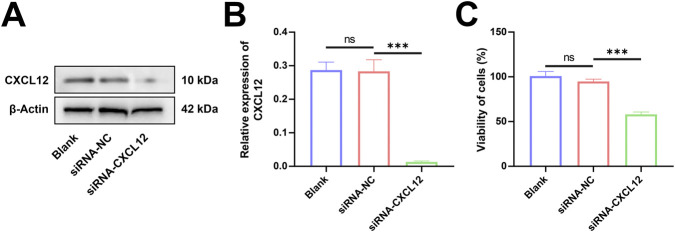
Silencing of CXCL12 inhibits cell growth. **(A)** Western blot analysis of CXCL12 expression in HTR-8 cells transfected with CXCL12 siRNA. **(B)** Statistical analysis performed after repeated experiments showed that the CXCL12 protein expression level of siRNA-CXCL12 was significantly lower than that of siRNA-NC. **(C)** The growth and proliferation of HTR-8 cells transfected by CXCL12 siRNA were significantly lower compared with siRNA-NC. Ns, no significance; ***p < 0.001. Blank: blank control group; siRNA-NC: empty control group; siRNA-CXCL12: CXCL12 silencing group.

HTR-8 cells were transfected with siRNA targeting the CXCL12 gene sequence, and Western blot analysis confirmed that CXCL12 expression was significantly silenced (Figure 5A). Statistical analysis after repeated experiments showed that CXCL12 was significantly under-expressed in HTR-8 cells after transfection with CXCL12 siRNA (Figure 5B, *p* < 0.001). The growth and proliferation of CXCL12-silenced HTR-8 cells were evaluated by MTT assay. The silencing of CXCL12 significantly inhibited the growth capacity of HTR-8 cells compared with the no-load control (Figure 5C, *p* < 0.001).

### Silencing CXCL12 suppresses the migratory and invasive capacity of cells

**FIGURE 6:**
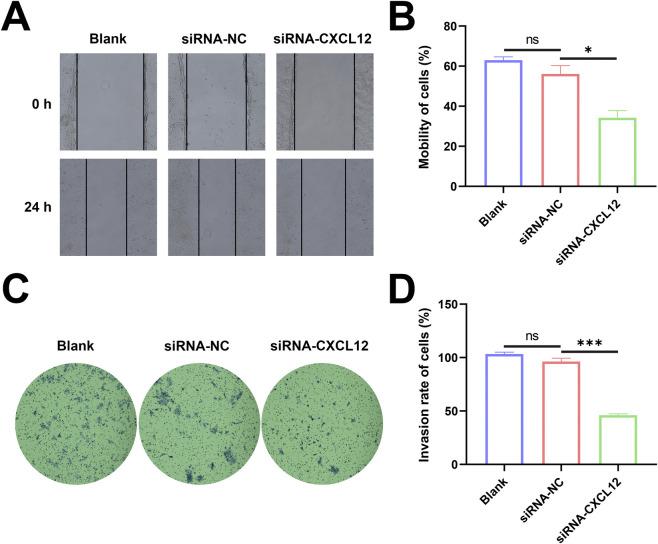
Silencing CXCL12 suppresses the migratory and invasive capacity of cells. **(A)** HTR-8 cells after CXCL12 silencing (100×). **(B)** The 24-h cell mobility decreased significantly after CXCL12 silencing compared with siRNA-NC2 (*p* < 0.05). **(C)** Invasion of HTR-8 cells invasion after CXCL12 silencing based on transwell assay (100×). **(D)** The 24-h cell mobility decreased significantly after CXCL12 silencing compared with siRNA-NC. Ns, no significance; *p < 0.05; ***p < 0.001. Blank: blank control group; siRNA-NC: empty control group; siRNA-CXCL12: CXCL12-silencing group.

The changes in horizontal migration ability of HTR-8 cells after CXCL12 silencing were determined using cell scratch assay. The de-expression of CXCL12 inhibited the horizontal migration ability of HTR-8 cells (Figure 6A), and the cell mobility in the CXCL12-silenced group was significantly decreased at 24 h after cell scratching (Figure 6B, *p* < 0.05). Based on transwell assay, the silencing of CXCL12 significantly inhibited the invasion of HTR-8 cells (Figure 6C), and the aggressiveness of CXCL12-silenced HTR-8 cells decreased compared with the no-load control (Figure 6D, *p* < 0.001). These results demonstrate that silencing CXCL12 inhibits the migratory and invasive ability of HTR-8 cells.

### CXCL12 promotes the decidualization of the endometrium

**FIGURE 7:**
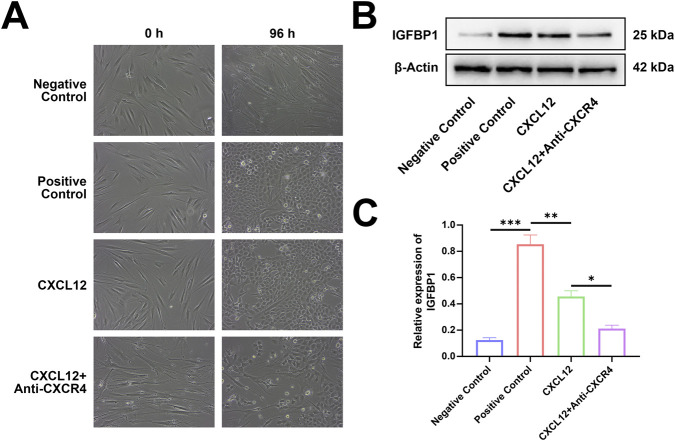
CXCL12 promotes the decidualization of the endometrium. **(A)** Recombinant human CXCL12 was used to stimulate ESCs, and the decidualization process was observed. **(B)** Expression levels of IGFBP-1 relative to β-actin in the four experimental groups (200×). **(C)** Endometrial stromal cells were stimulated by recombinant human CXCL12, the expression level of IGFBP-1 protein increased significantly. *p < 0.05; **p < 0.01; ***p < 0.001. Negative control: negative control group, no exogenous stimuli were added; Positive control: positive control group, medroxyprogesterone an 8-bromo-cAMP were added to stimulate the decidualization of ESCs; CXCL12: CXCL12 stimulation group, recombinant human CXCL12 was added as the stimulus; CXCL12+Anti-CXCR4, CXCL12 stimulation + CXCR4 neutralization group, recombinant human CXCL12 and CXCR4 neutralization antibody were added.

To test the role of CXCL12 in the endometrial decidualization process, we used recombinant human CXCL12 to stimulate ESCs and observed their decidualization process (Figure 7A). On day 4 after stimulation, the ESCs were transformed from fibroblasts to oval-shaped or even round DSCs. The marker of decidualization, IGFBP-1, was detected by Western blotting (Figure 7B). The expression level of IGFBP-1 was significantly higher in the CXCL12 stimulation group than in the negative control group (Figure 7C). To further verify the role of CXCL12, we added CXCR4-neutralizing antibody to the CXCL12-stimulated cells, which significantly decreased the degree of decidualization (Figure 7A). Western blot analysis confirmed that the expression level of IGFBP-1 in the CXCL12+Anti-CXCR4 group was significantly lower than that in the CXCL12 stimulation, although it was still higher than the expression in the negative control group (Figure 7C). These findings demonstrate that CXCL12 significantly promotes the decidualization process of the endometrium, and this effect can be partly reversed by CXCR4-neutralizing antibodies.

## Discussion

In mammals, embryo implantation into the uterus predicts the onset of pregnancy and initiates a complex series of conversations between the mother and fetus. These intimate interactions involve the specialized trophoblast cells of the embryo and the maternal permissive uterus. Steroid hormones including estrogen and progesterone play a key role in guiding uterine changes in early pregnancy. The successful implantation, growth, and survival of partially allogenic transplanted embryos in the maternal uterus depend on the integrity of the maternal-fetal interface established by fetal trophoblast cells and the maternal decidua. The main source of CXCL12 in early pregnancy is trophoblast cells. In the finely regulated chemokine network, the high expression of CXCL12 is conducive to the maintenance of pregnancy, and CXCL12 plays a positive role in both trophoblast and decidual cells. Abnormal interactions at the maternal-fetal interface will disrupt the biological function of multiple cells at the maternal-fetal interface; these disruptions are correlated with problems such as failed embryo implantation, abortion, premature birth, preeclampsia, and fetal functional growth restriction (Cecati et al., 2011). However, due to the complexity of the maternal-fetal interface and the limitations of current experimental methods, much remains unknown about the specialized mechanisms concerning the dialogue between the decidua and the embryo during normal pregnancy. In particular, the specific regulatory mechanism between trophoblast cell proliferation, migration, and invasion and endometrial decidualization in the maintenance of normal pregnancy remains unclear. Few studies have been conducted on CXCL12-related pathways; thus, we carried out related research.

Based on the GEO database and utilizing bioinformatics analysis methods, we identified single-cell atlas at the maternal fetal interface in normal and RM samples. Subsequently, we comparably analyzed the expression of CXCL12 of SCT/EVT in normal and unexplained RM pregnancies at the early stage of gestation. CXCL12 in SCT/EVT cells was remarkably decreased in RM cases. To explore the potential functional consequences of this downregulation, we performed functional enrichment analysis on differentially expressed genes. The analysis revealed broad involvement in RNA processing and metabolism, key pathways directly pertinent to the observed cellular phenotypes were significantly enriched. Specifically, genes downregulated in RM were notably enriched in the Cell cycle and sister chromatid segregation, providing a transcriptional clue that CXCL12 deficiency might hamper trophoblast proliferation. Furthermore, enrichment in the cadherin binding suggested a potential impact on cell-cell adhesion and motility, processes fundamental to migration and invasion (Wong et al., 2018).

Building on these bioinformatic insights, we constructed models of CXCL12 overexpression and silencing in trophoblast cells and examined the influence of CXCL12 on the biological characteristics of trophoblast cells. We found that CXCL12 significantly promoted the proliferation, migration, and invasion of trophoblast cells, supporting that CXCL12 plays a positive role in the course of pregnancy. It is widely accepted that the maternal-fetal interface is characterized by a complex cytokine network; the specific biological functions of the chemokines and their receptors in this network have received considerable attention. CXCL12 and CXCR4 are a specialized chemokine/chemokine receptor pair that plays important roles during pregnancy. The CXCL12/CXCR4 axis is involved in important biological processes such as the recruitment of uterine NK cells, placenta formation, embryo implantation, and embryogenesis (Santoni et al., 2008). The CXCL12/CXCR4 axis not only affects the activation, migration, and recruitment of immune cells, it also participates in processes such as angiogenesis, hematopoiesis, embryonic development, and tumor invasion and metastasis (Hunger et al., 2012; Sánchez-Martín et al., 2013; Tripathi et al., 2014). Trophoblast apoptosis is known to occur in the placenta during pregnancy and plays an important regulatory role in the migration of trophoblast cells and during placenta implantation (Liu et al., 2015). Although the exact mechanism of apoptosis in human trophoblast cells remains unclear, many different models for the induction and prevention of apoptosis have been proposed (Bolnick et al., 2011). Ren (Ren et al., 2012) confirmed that the membranes of human early pregnancy DSCs express CXCR4, and that trophoblast-derived CXCL12 can promote CXCR4 expression and the invasive ability of DSCs by linking to CXCR4. These findings highlight the synergistic role of the CXCL12/CXCR4 axis in both trophoblast cells and DSCs. Zhou (Zhou et al., 2008) demonstrated that trophoblast cells regulate their own function through an autocrine signaling mechanism by co-expressing CXCL12 and CXCR4. Meanwhile, trophoblast cells communicate with DSCs expressing CXCR4 through CXCL12-mediated paracrine signaling, which promotes matrix metalloproteinase activity, extracellular matrix degradation, and trophoblast invasion. Consistent with these findings, our experiments confirmed that CXCL12 promotes the proliferation, migration and invasion of trophoblast cells. Correspondingly, silencing CXCL12 limited these processes. Additionally, the observed low CXCL12 expression in villous tissue weakens its interaction with CXCR4 in decidual tissue. Beyond its direct effects on trophoblast and decidual cells, this CXCL12 deficiency may critically impair the establishment of a balanced immune microenvironment at the maternal-fetal interface. The CXCL12/CXCR4 axis is a well-established chemotactic signal for various immune cells. Specifically, it is pivotal for the recruitment and positioning of uterine natural killer (uNK) cells, particularly the CD56^+^ bright subset, and decidual macrophages (dMΦ) to the decidua, as demonstrated in both human and murine studies (Lu et al., 2020; Fang et al., 2022). uNK cells are essential for spiral artery remodeling and immune tolerance, while dMΦ play key roles in tissue remodeling and the clearance of apoptotic cells (Fang et al., 2022). Therefore, insufficient CXCL12 signaling may lead to inadequate or altered recruitment of these immune cells, potentially resulting in impaired vascular remodeling, dysregulated inflammatory responses, and a breakdown in fetal-maternal tolerance (Ao et al., 2020). This potential imbalance in the immune milieu, coupled with the direct impairment of trophoblast invasion and decidualization, could synergistically contribute to the pathogenesis of pregnancy loss. Of course, this also requires us to do in-depth verification in subsequent research.

Next, we investigated the effect of CXCL12 on the decidualization of ESCs *in vitro*. We found that CXCL12 significantly promoted the decidualization of ESCs, while this effect was partially reversed by the addition of CXCR4-neutralizing antibodies. Infertility due to impaired uterine function is a serious health problem among women and needs to be further explored. The action of estrogen and progesterone through their cognate receptors is essential for the accurate and timely regulation of the endometrial time required for pregnancy. The embryonic attachment, decidualization, and ultimate vascularization of the endometrium require the combined action of estrogen and progesterone along with the precise regulation of a range of signaling molecules. Increasing evidence suggests that both biochemical and metabolic factors play important roles in decidual formation. Lipid mediators such as lysophosphatidic acid are produced by the uterine epithelium (Aikawa et al., 2017) and regulate heparin-binding epidermal growth factor (Chobotova et al., 2005), epidermal growth factor receptor signaling, and cyclooxygenase-2 (Lim et al., 1997). This regulation leads to the production of prostaglandin E2 (Milne et al., 2001), which, together with interferon-γ (Christian et al., 2001), controls the decidualization of ESCs. Decidualization is regulated by various molecular mechanisms in addition to maternal hormones, biochemical factors, and metabolic signals. During decidualization, differentiated ESCs carry the molecular signature of the mesenchymal-epithelial transition; the ESCs are reconstituted into DSCs and undergo extensive changes in gene expression, including the induction of HOXA10, HOXA11, FOXO 1, WNT 4, IGFBP1, and prolactin (Zhang XH. et al., 2013; Owusu-Akyaw et al., 2019; Liu and Wang, 2015; Popovici et al., 2006). Many of these factors are upstream regulators of genes known to be critical for implantation and embryonic development (Harris et al., 2019). The decidualized cells, which have unique biosynthetic and secretory properties and exhibit substantial changes in the extracellular matrix, are a prerequisite for successful implantation. The decidua supports the formation of maternal blood vessels, which irrigate and nourish the developing embryo. Many complications observed in early pregnancy originate from abnormalities in implantation and placental development (Brosens et al., 2019). Although the functional importance of decidualization in human pregnancy is not fully understood, it appears to promote active embryonic implantation (Gellersen et al., 2010), negative selection of nonviable embryos (Teklenburg et al., 2010), determination of the optimal implant time window (Cha et al., 2012), and uterine hemostasis (Schatz et al., 2016). Recurrent spontaneous abortion has been associated with the dysregulation of decidualization markers, including prolactin, proagonin, and the DIO two and SCARA5 genes (Lucas et al., 2020), thereby impairing maternal periodic decidualization. Despite exhaustive studies on the molecular and cellular mechanisms of embryo implantation and placentation, interventions to prevent these complications have been mostly unsuccessful. In this study, we found that using recombinant CXCL12 to stimulate ESC significantly promoted the decidualization of ESCs, and this effect was blocked by CXCR4-neutralizing antibodies. Our results indicate a functional role for CXCL12 and CXCR4 in ESC decidualization under our experimental conditions. CXCL12 has also been shown to promote CXCR4 expression in DSCs (Mao et al., 2017). CXCL12 secreted by trophoblasts promotes the migration and invasion of DSCs (Mao et al., 2017; Benedicto et al., 2018). Our study also found that the process of decidualization was partially inhibited after the addition of neutralizing antibodies of CXCR4, suggesting that CXCL12 may also contribute to decidualization progression of ESC through other mechanisms. This is consistent with the findings of our previous study: the transcript level of CXCR4 was not different in the decidual tissues of women with miscarriages and normal pregnancies, although miscarriage was associated with decreased protein expression level (Sun et al., 2022). This suggests a possibility that the low expression of CXCR4 in decidual tissue is caused by the inability of trophoblast cells to secrete sufficient CXCL12, which could further affecting the migration and invasion of DSCs and potentially the process of endometrial decidualization, thereby constituting a potential risk factor for abortion.

However, our research has many limitations. (1) The sample size of scRNA-seq analysis is small and the population source is single. In the future, it is necessary to verify based on larger prospective clinical samples and multi-center data to further enhance the extrapolation of research conclusions in a wider population. (2) However, due to the lack of detailed and standardized clinical metadata in the currently available scRNA-seq data set, the association between CXCL12 expression levels and key clinical parameters (such as gestational age, number of previous abortions, serum hormone status, and pregnancy outcome) was not further explored. Such clinical correlation analysis is of great clinical significance because they may help to determine whether CXCL12 can not only be used as a mechanism molecule, but also as a potential biomarker for abortion risk stratification, early diagnosis and prognosis evaluation. In the future, it is necessary to systematically study the threshold, sensitivity and specificity of CXCL12 at the transcriptional and protein levels based on large-scale, well-annotated clinical cohorts and prospective data collection, and to clarify its potential application value in clinical decision-making. (3) This study is mainly based on *in vitro* models and cannot fully reproduce the complexity of microenvironment and systemic regulation in RM. Therefore, the precise causal relationship and clinical transformation potential of CXCL12 need to be verified in future *in vivo* and clinical cohort studies. (4) Although our data show that CXCL12 can enhance the decidualization of endometrial stromal cells, and CXCR4 neutralizing antibody can attenuate this effect, its exact signal transduction mechanism remains to be fully elucidated. In addition to acting through the classic CXCR4 receptor, CXCL12 may also act through other potential mediators, such as alternative receptor CXCR7 (ACKR3), or activation of downstream signaling cascades, such as PI3K/AKT and MAPK/ERK, which are usually associated with cell differentiation and survival, and may also be involved. Future studies should use receptor-specific knockdown (such as CXCR4 or CXCR7) combined with phosphorylated protein analysis of these key signaling pathways to dissect the exact contribution of each component to CXCL12-mediated decidualization. In conclusion, our findings demonstrate that CXCL12 promotes the proliferation, migration, and invasion of trophoblast cells as well as the decidualization of ESCs. The downregulation of CXCL12 observed in RM samples, coupled with these functional effects, suggests that impaired CXCL12 signaling may be associated with pregnancy loss. Therefore, CXCL12 represents a potential target for further investigation into the mechanisms underlying threatened abortion. This provides direction for subsequent research.

## Data Availability

The datasets presented in this study can be found in online repositories. The names of the repository/repositories and accession number(s) can be found in the article/supplementary material.

## References

[B1] Aikawa S. Kano K. Inoue A. Wang J. Saigusa D. Nagamatsu T. (2017). Autotaxin-lysophosphatidic acid-LPA(3) signaling at the embryo-epithelial boundary controls decidualization pathways. EMBO Journal 36 (14), 2146–2160. 10.15252/embj.201696290 28588064 PMC5509998

[B2] Ao D. Li D. J. Li M. Q. (2020). CXCL12 in normal and pathological pregnancies: a review. Am. Journal Reproductive Immunol. N. Y. N. Y. 84 (3), e13280. 10.1111/aji.13280 32485053

[B3] Benedicto A. Romayor I. Arteta B. (2018). CXCR4 receptor blockage reduces the contribution of tumor and stromal cells to the metastatic growth in the liver. Oncol. Reports 39 (4), 2022–2030. 10.3892/or.2018.6254 29436696

[B4] Bhurke A. S. Bagchi I. C. Bagchi M. K. (2016). Progesterone-regulated endometrial factors controlling implantation. Am. Journal Reproductive Immunology New York, N. Y. 75 (3), 237–245. 10.1111/aji.12473 26804062 PMC4754106

[B5] Bolnick J. Albitar L. Laidler L. L. Abdullah R. Leslie K. K. (2011). Blocking epidermal growth factor receptor signaling in HTR-8/SVneo first trimester trophoblast cells results in dephosphorylation of PKBα/AKT and induces apoptosis. Obstetrics Gynecology International 2011, 896896. 10.1155/2011/896896 21876698 PMC3159379

[B6] Brosens I. Puttemans P. Benagiano G. (2019). Placental bed research: I. The placental bed: from spiral arteries remodeling to the great obstetrical syndromes. Am. Journal Obstetrics Gynecology 221 (5), 437–456. 10.1016/j.ajog.2019.05.044 31163132

[B7] Caballero-Campo P. Domínguez F. Coloma J. Meseguer M. Remohí J. Pellicer A. (2002). Hormonal and embryonic regulation of chemokines IL-8, MCP-1 and RANTES in the human endometrium during the window of implantation. Mol. Human Reproduction 8 (4), 375–384. 10.1093/molehr/8.4.375 11912286

[B8] Cecati M. Giannubilo S. R. Emanuelli M. Tranquilli A. L. Saccucci F. (2011). HLA-G and pregnancy adverse outcomes. Med. Hypotheses 76 (6), 782–784. 10.1016/j.mehy.2011.02.017 21376476

[B9] Cha J. Hirota Y. Dey S. K. (2012). Sensing senescence in preterm birth. Cell CycleGeorget. Tex 11 (2), 205–206. 10.4161/cc.11.2.18781 22189716

[B10] Chobotova K. Karpovich N. Carver J. Manek S. Gullick W. J. Barlow D. H. (2005). Heparin-binding epidermal growth factor and its receptors mediate decidualization and potentiate survival of human endometrial stromal cells. J. Clinical Endocrinology Metabolism 90 (2), 913–919. 10.1210/jc.2004-0476 15562026 PMC1626580

[B11] Christian M. Marangos P. Mak I. McVey J. Barker F. White J. (2001). Interferon-gamma modulates prolactin and tissue factor expression in differentiating human endometrial stromal cells. Endocrinology 142 (7), 3142–3151. 10.1210/endo.142.7.8231 11416037

[B12] Fang Y. Y. Lyu F. Abuwala N. Tal A. Chen A. Y. Taylor H. S. (2022). Chemokine C-X-C receptor 4 mediates recruitment of bone marrow-derived nonhematopoietic and immune cells to the pregnant uterus. Biol. Reproduction 106 (6), 1083–1097. 10.1093/biolre/ioac029 35134114 PMC9198949

[B13] Gellersen B. Reimann K. Samalecos A. Aupers S. Bamberger A. M. (2010). Invasiveness of human endometrial stromal cells is promoted by decidualization and by trophoblast-derived signals. Hum. Reproduction Oxf. Engl. 25 (4), 862–873. 10.1093/humrep/dep468 20118488

[B14] Hanna J. Wald O. Goldman-Wohl D. Prus D. Markel G. Gazit R. (2003). CXCL12 expression by invasive trophoblasts induces the specific migration of CD16-human natural killer cells. Blood 102 (5), 1569–1577. 10.1182/blood-2003-02-0517 12730110

[B15] Hannan N. J. Salamonsen L. A. (2007). Role of chemokines in the endometrium and in embryo implantation. Curr. Opinion Obstetrics and Gynecology 19 (3), 266–272. 10.1097/GCO.0b013e328133885f 17495644

[B16] Hanson B. Johnstone E. Dorais J. Silver B. Peterson C. M. Hotaling J. (2017). Female infertility, infertility-associated diagnoses, and comorbidities: a review. J. Assisted Reproduction Genetics 34 (2), 167–177. 10.1007/s10815-016-0836-8 27817040 PMC5306404

[B17] Harris L. K. Benagiano M. D'Elios M. M. Brosens I. Benagiano G. (2019). Placental bed research: II. Functional and immunological investigations of the placental bed. Am. Journal Obstetrics Gynecology 221 (5), 457–469. 10.1016/j.ajog.2019.07.010 31288009

[B18] Hendriks E. MacNaughton H. MacKenzie M. C. (2019). First trimester bleeding: evaluation and management. Am. Family Physician 99 (3), 166–174. 30702252

[B19] Hunger C. Ödemis V. Engele J. (2012). Expression and function of the SDF-1 chemokine receptors CXCR4 and CXCR7 during mouse limb muscle development and regeneration. Exp. Cell Research 318 (17), 2178–2190. 10.1016/j.yexcr.2012.06.020 22766125

[B20] Koopman L. A. Kopcow H. D. Rybalov B. Boyson J. E. Orange J. S. Schatz F. (2003). Human decidual natural killer cells are a unique NK cell subset with immunomodulatory potential. J. Experimental Medicine 198 (8), 1201–1212. 10.1084/jem.20030305 14568979 PMC2194228

[B21] Lim H. Paria B. C. Das S. K. Dinchuk J. E. Langenbach R. Trzaskos J. M. (1997). Multiple female reproductive failures in cyclooxygenase 2-deficient mice. Cell 91 (2), 197–208. 10.1016/s0092-8674(00)80402-x 9346237

[B22] Liu J. L. Wang T. S. (2015). Systematic analysis of the molecular mechanism underlying decidualization using a text mining approach. PloS One 10 (7), e0134585. 10.1371/journal.pone.0134585 26222155 PMC4519252

[B23] Liu X. Dai L. I. Zhou R. (2015). Association between preeclampsia and the CXC chemokine family. Exp. Therapeutic Medicine 9 (5), 1572–1576. 10.3892/etm.2015.2337 26136860 PMC4471657

[B24] Lu H. Jin L.-P. Huang H.-L. Ha S.-Y. Yang H.-L. Chang R.-Q. (2020). Trophoblast‐derived CXCL12 promotes CD56brightCD82−CD29+ NK cell enrichment in the decidua. Am. J. Reprod. Immunol. 83. 10.1111/aji.13203 31650642

[B25] Lucas E. S. Vrljicak P. Muter J. Diniz-da-Costa M. M. Brighton P. J. Kong C. S. (2020). Recurrent pregnancy loss is associated with a pro-senescent decidual response during the peri-implantation window. Commun. Biology 3 (1), 37. 10.1038/s42003-020-0763-1 31965050 PMC6972755

[B26] Mao T. L. Fan K. F. Liu C. L. (2017). Targeting the CXCR4/CXCL12 axis in treating epithelial ovarian cancer. Gene Therapy 24 (10), 621–629. 10.1038/gt.2017.69 28753202

[B27] Milne S. A. Perchick G. B. Boddy S. C. Jabbour H. N. (2001). Expression, localization, and signaling of PGE(2) and EP2/EP4 receptors in human nonpregnant endometrium across the menstrual cycle. J. Clinical Endocrinology Metabolism 86 (9), 4453–4459. 10.1210/jcem.86.9.7856 11549693

[B28] Owusu-Akyaw A. Krishnamoorthy K. Goldsmith L. T. Morelli S. S. (2019). The role of mesenchymal-epithelial transition in endometrial function. Hum. Reproduction Update 25 (1), 114–133. 10.1093/humupd/dmy035 30407544

[B29] Piao H. L. Tao Y. Zhu R. Wang S. C. Tang C. L. Fu Q. (2012). The CXCL12/CXCR4 axis is involved in the maintenance of Th2 bias at the maternal/fetal interface in early human pregnancy. Cell. and Molecular Immunology 9 (5), 423–430. 10.1038/cmi.2012.23 22885527 PMC4002329

[B30] Popovici R. M. Betzler N. K. Krause M. S. Luo M. Jauckus J. Germeyer A. (2006). Gene expression profiling of human endometrial-trophoblast interaction in a coculture model. Endocrinology 147 (12), 5662–5675. 10.1210/en.2006-0916 16946011

[B31] Red-Horse K. Kapidzic M. Zhou Y. Feng K. T. Singh H. Fisher S. J. (2005). EPHB4 regulates chemokine-evoked trophoblast responses: a mechanism for incorporating the human placenta into the maternal circulation. Dev. Camb. Engl. 132 (18), 4097–4106. 10.1242/dev.01971 16107476

[B32] Ren L. Liu Y. Q. Zhou W. H. Zhang Y. Z. (2012). Trophoblast-derived chemokine CXCL12 promotes CXCR4 expression and invasion of human first-trimester decidual stromal cells. Hum. Reproduction Oxf. Engl. 27 (2), 366–374. 10.1093/humrep/der395 22114110

[B33] Salamonsen L. A. Hannan N. J. Dimitriadis E. (2007). Cytokines and chemokines during human embryo implantation: roles in implantation and early placentation. Seminars Reproductive Medicine 25 (6), 437–444. 10.1055/s-2007-991041 17960528

[B34] Sánchez-Martín L. Sánchez-Mateos P. Cabañas C. (2013). CXCR7 impact on CXCL12 biology and disease. Trends Molecular Medicine 19 (1), 12–22. 10.1016/j.molmed.2012.10.004 23153575

[B35] Santoni A. Carlino C. Gismondi A. (2008). Uterine NK cell development, migration and function. Reprod. Biomedicine Online 16 (2), 202–210. 10.1016/s1472-6483(10)60575-5 18284874

[B36] Schanz A. Winn V. D. Fisher S. J. Blumenstein M. Heiss C. Hess A. P. (2011). Pre-eclampsia is associated with elevated CXCL12 levels in placental syncytiotrophoblasts and maternal blood. Eur. Journal Obstetrics, Gynecology, Reproductive Biology 157 (1), 32–37. 10.1016/j.ejogrb.2011.02.023 21450389

[B37] Schatz F. Guzeloglu-Kayisli O. Arlier S. Kayisli U. A. Lockwood C. J. (2016). The role of decidual cells in uterine hemostasis, menstruation, inflammation, adverse pregnancy outcomes and abnormal uterine bleeding. Hum. Reproduction Update 22 (4), 497–515. 10.1093/humupd/dmw004 26912000 PMC4917742

[B38] Sun X. L. Zhao J. Leng Z. Lin H. Huang Y. (2022). Low expression levels of CXCL12, CXCR4, and CXCR 7 in peripheral blood and decidual tissues are associated with miscarriage in women. Immunol. Investigations 51 (7), 2053–2065. 10.1080/08820139.2022.2106871 35912820

[B39] Tamrakar S. R. Bastakoti R. (2019). Determinants of infertility in couples. J. Nepal Health Res. Counc. 17 (1), 85–89. 10.33314/jnhrc.1827 31110383

[B40] Teklenburg G. Salker M. Molokhia M. Lavery S. Trew G. Aojanepong T. (2010). Natural selection of human embryos: decidualizing endometrial stromal cells serve as sensors of embryo quality upon implantation. PloS One 5 (4), e10258. 10.1371/journal.pone.0010258 20422011 PMC2858159

[B41] Tripathi V. Kumar R. Dinda A. K. Kaur J. Luthra K. (2014). CXCL12-CXCR7 signaling activates ERK and Akt pathways in human choriocarcinoma cells. Cell Communication and Adhesion 21 (4), 221–228. 10.3109/15419061.2013.876013 24450273

[B42] Wei P. Dong M. Bi Y. Chen S. Huang W. Li T. (2022). Identification and validation of a signature based on macrophage cell marker genes to predict recurrent miscarriage by integrated analysis of single-cell and bulk RNA-sequencing. Front. Immunology 13, 1053819. 10.3389/fimmu.2022.1053819 36439123 PMC9692009

[B43] Wong S. H. M. Fang C. M. Chuah L. H. Leong C. O. Ngai S. C. (2018). E-cadherin: its dysregulation in carcinogenesis and clinical implications. Crit. Reviews Oncology/Hematology. 121, 11–22. 10.1016/j.critrevonc.2017.11.010 29279096

[B44] Wu X. Li D. J. Yuan M. M. Zhu Y. Wang M. Y. (2004). The expression of CXCR4/CXCL12 in first-trimester human trophoblast cells. Biol. Reproduction 70 (6), 1877–1885. 10.1095/biolreprod.103.024729 14973260

[B45] Wu X. Jin L. P. Yuan M. M. Zhu Y. Wang M. Y. Li D. J. (2005). Human first-trimester trophoblast cells recruit CD56brightCD16- NK cells into decidua by way of expressing and secreting of CXCL12/stromal cell-derived factor 1. J. Immunology Baltim. 175 (1), 61–68. 10.4049/jimmunol.175.1.61 15972632

[B46] Zhang S. Lin H. Kong S. Wang S. Wang H. Wang H. (2013a). Physiological and molecular determinants of embryo implantation. Mol. Aspects Medicine 34 (5), 939–980. 10.1016/j.mam.2012.12.011 23290997 PMC4278353

[B47] Zhang X. H. Liang X. Liang X. H. Wang T. S. Qi Q. R. Deng W. B. (2013b). The mesenchymal-epithelial transition during *in vitro* decidualization. Reprod. Sciences Thousand Oaks, Calif. 20 (4), 354–360. 10.1177/1933719112472738 23302397 PMC4077516

[B48] Zheng J. Wang H. Zhou W. (2018). Modulatory effects of trophoblast-secreted CXCL12 on the migration and invasion of human first-trimester decidual epithelial cells are mediated by CXCR4 rather than CXCR7. Reproductive Biology Endocrinology RB&E 16 (1), 17. 10.1186/s12958-018-0333-2 29499763 PMC5833108

[B49] Zhou W. H. Du M. R. Dong L. Yu J. Li D. J. (2008). Chemokine CXCL12 promotes the cross-talk between trophoblasts and decidual stromal cells in human first-trimester pregnancy. Hum. Reproduction Oxf. Engl. 23 (12), 2669–2679. 10.1093/humrep/den308 18687671

